# Telehealth and challenges of statin adherence in patients with diabetes: a randomized controlled trial

**DOI:** 10.1186/s12913-025-13295-3

**Published:** 2025-08-29

**Authors:** Amna A. Desoky, Neama M. Mostafa, Mohammad HM AbdEllah-Alawi, Eman M. Hashem

**Affiliations:** 1 Medical-Surgical Nursing, Faculty of Nursing, Assiut University, Assiut, Egypt; 2 Internal Medicine, Faculty of Medicine, Assiut University, Assiut, Egypt

**Keywords:** Adherence, Diabetes, Statin, Telehealth

## Abstract

**Background:**

The success of any therapeutic regimen can be achieved only if patients adhere to the prescribed regimen. Finding an effective intervention to improve patient adherence is challenging.

**Aim:**

To evaluate the effect of telehealth through short messaging services (SMSs) on adherence to statins in patients with diabetes.

**Methods:**

This was a randomized clinical trial. Patients with diabetes mellitus were referred to the Center of Diabetes and Endocrinology at Assiut University Hospital and were assessed for eligibility. Ninety participants were randomly assigned to either the intervention or control group. Reminder SMS messages were regularly sent to the intervention group. Follow-up was performed at 6 and 12 weeks. Medication Adherence Report Scale-5 (MARS-5) and pill counting (PC) were used to measure adherence. The lipid profile, incidence of acute cardiovascular events, and mortality were assessed as secondary outcomes.

**Results:**

After six weeks, both groups exhibited similar adherence rates based on the subjective MARS-5 score. However, the intervention group demonstrated significantly better PC adherence than the control group. By the 12-week follow-up, the intervention group showed modest but statistically significant improvements in adherence rates (MARS-5, PC), along with reductions in total cholesterol, LDL-c levels, morbidity, and mortality incidence during the second follow-up period. These improvements were consistently greater in the intervention group compared to the control group (*P* < 0.05).

**Conclusions:**

Telehealth through SMS has been shown to positively impact statin adherence in patients with diabetes, leading to an improved lipid profile, a reduced risk of acute cardiovascular events, and lower overall mortality.

**Trial registration:**

https://www.clinicaltrials.gov. Identifier: NCT 05872919 Date of registration July 15-2023.

**Supplementary Information:**

The online version contains supplementary material available at 10.1186/s12913-025-13295-3.

## Background

Telehealth is an innovative approach that utilizes information and communication technologies to deliver healthcare services remotely. The widespread availability of mobile phones presents an opportunity to implement behavior change interventions on a large scale at a relatively low cost. In low- and middle-income countries, mobile phone usage exceeds 90%, making mobile health (mHealth) interventions feasible in these regions [1] In Egypt, mobile subscriptions reached 106.2 million by December 2023. Additionally, the internet penetration rate reached 72.2%, indicating widespread digital connectivity across the country [2].

Diabetes mellitus (DM) is a leading global cause of death, arising from either insulin deficiency (Type 1) or resistance (Type 2). Type 2 DM accounts for most cases due to lifestyle-related risk factors. In Egypt, DM affects 22.4% of adults, with predictions indicating a rise to 24.7 million cases by 2050 [3]. Poorly controlled DM can result in serious complications and increased mortality [4].

Dyslipidemia, a key marker of endothelial dysfunction, significantly increases the risk of atherosclerosis and cardiovascular disease (CVD), especially when combined with diabetes. Although manageable through lifestyle changes and medications like statins, adherence to statin is often poor due to the lack of immediate effects. Preventive use of cost-effective medications, including statins, ACE inhibitors, beta-blockers, and antiplatelet agents, is essential to reduce cardiovascular events [5, 6]. 

Statins are recommended as the first-line therapy in most clinical guidelines due to their ability to lower C-reactive protein (CRP) levels, a key marker of arterial inflammation linked to heart attacks and strokes [7, 8] In Egypt, poor adherence to lipid-lowering therapy among patients with diabetes remains a significant challenge [9]. A systematic review and meta-analysis [10] highlighted that mobile phone text messaging interventions significantly improve medication adherence in patients with chronic conditions. While mobile health interventions have been explored for diabetes management [11, 12, 13] relatively few have specifically targeted statin adherence. This study addresses that gap by evaluating the effectiveness of SMS-based intervention to improve statin adherence in patients with diabetes.

### Study objectives and hypotheses

The current study aimed to evaluate the effect of telehealth through the SMS (Short Messaging Service) on adherence to lipid-lowering therapy among patients with diabetes as a primary outcome. Secondary outcomes included changes in clinical parameters. The main hypothesis proposed that patients who consistently received SMS reminders would demonstrate higher medication adherence at follow-up compared to those who did not receive such messages. A secondary hypothesis suggested that improved adherence would be associated with improved lipid profiles, reduced risk of acute cardiovascular events, and mortality.

## Methods

### Design

**Fig. 1:**
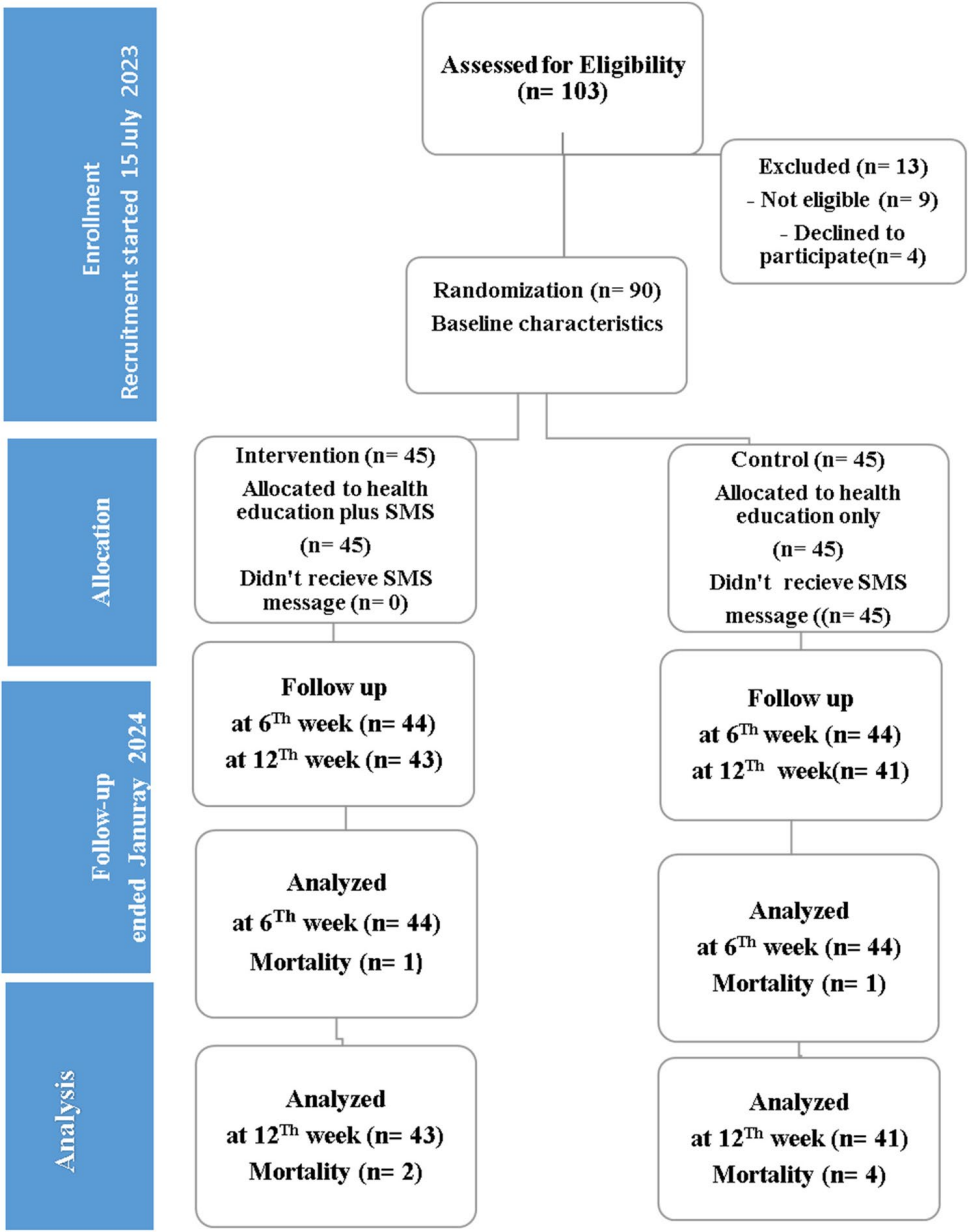
Consort diagram of the flow of participants through the study

The true experimental design was a “randomized controlled trial”. The study was approved by the “Research Ethics Committee” of the Faculty of Nursing, Assiut University (IRB: 1120240473), in accordance with the World Medical Association Declaration of Helsinki Ethical Principles for Medical Research Involving Human Subjects. Informed consent was obtained from all patients prior to their participation in the study. “The findings of this study are presented in accordance with the Consolidated Standards of Reporting Trials (CONSORT) 2010 guidelines [14]. A summary of the study design is illustrated in Fig. 1."

### Setting

This study was conducted at the Center of Diabetes and Endocrinology, Assiut University Hospital.

### Pilot study

Before data collection, a pilot study was conducted on 10 participants to assess the clarity, feasibility, and applicability of the study tools and to estimate the time required for data collection. Necessary modifications were made, such as using a clearer, shorter, and simpler Arabic for SMS. The pilot study sample was excluded from the main study sample.

### Participants

The study population included patients with diabetes who were already prescribed lipid-lowering medications. The sample size was calculated to be at least 42 participants based on previous study [11], as 68% of the participants in the study group had high adherence, while 35.5% of the participants in the control group had low adherence, with an effect size of 0.49, a statistical power of 90%, a confidence level of 95%, an alpha of 0.05, and a beta of 0.1, considering 10% sample attrition (4 patients).

A total of 103 patients were initially screened for eligibility. 13 were excluded- 9 due to ineligibility (including coronary heart disease, communication difficulties, and psychotropic medication use) and 4 who declined participation. The remaining 90 participants were randomly assigned to either the intervention or control group (*n* = 45 each). Eligibility criteria required 18 years or older, diagnosed with type 1 or 2 diabetes, currently prescribed lipid-lowering medication for at least two weeks, have access to a mobile phone, capable of receiving SMS, and be able to read the messages independently or with help.

The control group received only health education, without any SMS reminders. One participant was lost to follow-up by the 6th week, and an additional three were lost by the 12th week (total of 4 deaths). All participants who remained available at each follow-up point were included in the corresponding analyses. The intervention group first received health education, followed by SMS reminders. All 45 participants in this group received the SMS intervention. During the follow-up period, one participant was lost by the 6th week, and another by the 12th week due to death, resulting in a total of two deaths.

### Randomization

After completing the baseline assessments, participants were assigned to either the intervention or control group using a simple randomization technique with an equal allocation ratio (1:1). An independent researcher—who was not involved in participant recruitment, outcome assessment, or data analysis—generated the random allocation sequence using a computerized random number generator (Microsoft Excel). To preserve allocation concealment, the assignments were placed in sequentially numbered, opaque, and sealed envelopes. These envelopes, prepared in advance based on the random sequence, were opened only after each participant had been enrolled and their baseline data collected. Group labels were coded as ‘I’ and ‘II’ to avoid revealing group identities during the study.

Blinding was rigorously maintained throughout the trial. The dependent researchers, laboratory personnel responsible for evaluating outcomes, and the statistical analyst were all blinded to group assignments. Participants were also instructed not to disclose any information that might reveal their assigned group during follow-up visits. The actual group codes were only revealed after completion of all data analyses to ensure the integrity of the blinding process.

### Study instrument

Data collection instrument consisted of four parts. The first part included demographic and clinical data, the second included laboratory investigations. The third part featured the Morisky Medication Adherence Rate Scale (MARS-5) [7]and the fourth assessed acute cardiovascular events, including angina, myocardial infarction, cerebrovascular accidents, and mortality.

### Procedure

All participants underwent a comprehensive health history assessment, including specific questions regarding polypharmacy (defined as the use of five or more medications) [15]current lipid-lowering medication, drug type, dosage, frequency, and any side effects. Physical examination was conducted, including anthropometric measurements, waist circumference was measured at the midpoint between the lateral iliac crest and the lowest rib. The researcher also recorded the number of prescribed tablets.

Laboratory assessments included HbA1c and lipid profile measurements. Patients were asked to undergrow recent laboratory tests during follow-up. Since fasting is not required for cholesterol testing, non-fasting blood samples were sufficient.

### Intervention

Both intervention and control groups received a health education session after baseline assessment. Session designed to provide essential information for maintaining a healthy lipid profile. Using face-to-face methods, patients were educated on key topics, including adopting a low-fat diet, reducing saturated fat intake from meats and dairy products, engaging in regular aerobic exercise, and maintaining a healthy lifestyle. Additionally, the session emphasized the importance of medication adherence, outlining the benefits of lipid-lowering drugs and the potential consequences of non-adherence, as well as the necessity of adhering to other prescribed treatments.

The intervention group received personalized SMS reminders, which were prepared and sent by independent researcher under the oversight of the study coordinator. The initial message was sent via mobile phone on the second day following recruitment, using a template such as: “Mr./Ms. (patient name), it is time to take (medication name), (dose), (number of tablets) at (time).” These messages were tailored using information from the patients’ medical records. Participants were also asked to provide two alternative phone numbers to verify receipt of the messages. SMS reminders were delivered daily in the evening for the first six weeks, then reduced to three times per week for the subsequent six weeks. The research assistant monitored delivery status and followed up using alternate contacts when receipt could not be confirmed.

### Outcomes

The primary outcome of the study was the adherence rate, it was assessed using two indirect methods: an objective measure through pill count (PC) and a subjective measure using the MARS-5 scale. PC was determined by calculating the total number of days covered by lipid-lowering medication divided by the number of days in the study period. The adherence index date was set on the day of patient recruitment. Patients were classified as having good adherence if their estimated PC was **≥** 80%, a widely accepted threshold based on previous research [16].

MARS-5 is a self-reported tool used to assess nonadherence behaviors, utilizing a five-point Likert scale ranging from 1 (always) to 5 (never). The total score ranges from 5 to 25, with higher scores reflecting better medication adherence. A score of 20 or below indicates poor adherence. The reliability of the scale is supported by a Cronbach’s alpha of 0.89 ^7^. Secondary outcomes included clinical measures such as glycated hemoglobin (HbA1c) levels, lipid profile, incidence or hospitalization due to acute cardiovascular events (CVEs), and/or mortality.

### Statistical analysis

Data entry and analysis were performed using SPSS version 22 (Statistical Package for Social Science). Data are presented as numbers, percentages, means, and standard deviations. Chi-square and Fisher Exact tests were used to compare qualitative variables. The independent sample t-test was used to compare quantitative variables between groups. The paired sample t-test was used to compare quantitative data between the pretest and posttest. P-value considered statistically significant when (*P* < 0.05).

## Results

### Baseline demographic and clinical data

All available participants were included in the respective analyses at each follow-up point. No dropouts occurred for reasons other than death. Participants were enrolled between mid-July and September 2023, follow-up completed by the end of December 2023. According to the demographic data, the results of the current study revealed that the participants were homogeneous. The mean age in the intervention group was 55.4 ± 8.5 years, while in the control group it was 53.8 ± 9.2 years (*P* = 0.400). A slightly higher proportion of participants were female in both groups. 53.3% in the intervention group and 51.1% in the control group (*P* = 0.833). Additionally, 62.2% of participants in both groups had government-provided health insurance coverage (*P* = 1.000) (Table 1).

**Table 1:** Baseline characteristics of the study participants

Variables	Intervention (*n* = 45)		Control (*n* = 45)		*P*-value
	No	%	No	%	
Demographic data					
Age in years	55.40 ± 8.53		53.82 ± 9.15		0.400
Gender (men)	21	46.70	22	48.90	0.833
Marital status(married)	42	93.30	44	97.80	0.616
Residence (rural)	39	86.70	37	82.20	0.561
Education(educated)	19	42.20	22	48.90	0.525
Occupation (work)	15	33.33	17	37.80	0.823
Health insurance (governmental)	28	62.20	28	62.20	1.000
Clinical data					
CWaist circumference Mean ± SD	107.76 ± 18.68		102.84 ± 18.31		0.211
Type of diabetes (Type 2)	42	93.30	44	97.80	0.616
Duration of diabetes (Years)	9.60 ± 7.20		10.04 ± 7.02		0.661
Hypertension	30	66.70	29	64.40	0.824
Renal (CKD)	3	6.70	5	11.10	0.347
Number of medications used per day (Polypharmacy)	7.76 ± 2.40		7.38 ± 3.11		0.745
Statin (alone)	39	86.70	38	84.40	0.764
Stain + ezetimibe	6	13.30	7	15.60	
Onset of statin administration:					
New user	30	66.70	21	46.70	0.056
Old user	15	33.30	24	53.30	
Lab investigations					
HbA1c	9.95 ± 2.92		9.34 ± 2.01		0.254
Total cholesterol	165.13 ± 67.53		175.76 ± 55.52		0.417
LDL-cholesterol	91.48 ± 55.46		96.97 ± 50.66		0.625
HDL-cholesterol	32.14 ± 12.49		29.92 ± 9.24		0.341

*CKD* chronic kidney disease, *HbA1c* Glycosylated hemoglobin A1c, *LDL* Low density lipoprotein, *HDL* High-density lipoprotein (*P* >0.05)

The clinical characteristics of participants were comparable between the two groups. More than 90% of participants in both the intervention and control groups had type 2 diabetes —93.3% and 97.8%, respectively (*P* = 0.616)- with a mean disease duration of approximately 9 years in the intervention group and 10 years in the control group (*P* = 0.661). Hypertension was present in 66.7% of the intervention group and 64.4% of the control group (*P* = 0.824). On average, participants used seven medications daily, indicating polypharmacy (*P* = 0.745), including lipid-lowering agents.

Statins were the only lipid-lowering drugs used, with some patients also receiving ezetimibe as an add-on therapy; in all cases, only one tablet per day was administered. Statin users were classified as either new users (initiated within the last 12 weeks) or long-term users. Laboratory findings indicated that both groups exhibited elevated HbA1c levels and abnormal lipid profiles, including increased total cholesterol and LDL, along with reduced HDL cholesterol. These differences were not statistically significant (*P* = 0.254, 0.417, 0.625, and 0.341, respectively), as presented in (Table 1).

### Intervention effects

#### Primary outcome (adherence rate)

During the initial six-weeks follow-up, there was no statistically significant difference in MARS-5 scores between the intervention and control groups (*P* = 0.290). However, by the second follow-up, the intervention group demonstrated a significant improvement in MARS-5 scores (24.23 ± 1.34) compared to the control group (19.00 ± 3.20) (*P* = 0.000). Furthermore, the pill count (PC) ratio showed a significant increase in the intervention group during both follow-up periods (98.11 ± 3.40 and 99.07 ± 2.75) compared to the control group (91.29 ± 6.19 and 90.41 ± 7.68) (*P* = 0.000), as detailed in (Table 2).

**Table 2:** Primary outcome of the study participants

Adherence rates	Intervention (*n* = 45)			Control (*n* = 45)		*P*-value
		No	%	No	%	
Morisky Medication Adherence Scale − 5 (MARS-5)						
1 st follow-up	Mean ± SD	21.07 ± 2.29		20.55 ± 2.98		0.358
	≥ 20	37	84.10	33	75.00	0.290
	< 20	7	15.90	11	25.00	
2nd follow-up	Mean ± SD	24.23 ± 1.34		19.00 ± 3.20		0.000*
	≥ 20	42	97.70	23	56.10	0.000*
	< 20	1	2.30	18	43.90	
Pill count (PC)						
1 st follow-up	Mean ± SD	98.11 ± 3.40		91.29 ± 6.19		0.000*
	≥ 80%	44	100.00	43	97.70	
	< 80%	0	0.00	1	2.30	
2nd follow-up	Mean ± SD	99.07 ± 2.75		90.41 ± 7.68		0.000*
	≥ 80%	43	100.00	38	92.70	
	< 80%	0	0.00	3	7.30	

Chi-square test

#### Secondary outcome (clinical outcome)

Table 3 summarizes the mean changes in serum lipid profiles at the second follow-up among patients who adhered to lipid-lowering therapy (LLT) after receiving SMS reminder messages. The intervention group demonstrated significantly greater reductions in total cholesterol (135.26 ± 52.62 vs. 186.85 ± 54.89; *P* = 0.000), LDL-c (71.09 ± 46.66 vs. 108.42 ± 51.93; *P* = 0.000), and higher HDL-c levels (39.86 ± 10.25 vs. 26.14 ± 8.15; *P* = 0.000) compared to the control group. Moreover, Fewer patients in the intervention group experienced acute cardiovascular events (18.6% vs. 34.1%; *P* = 0.105) and mortality (4.4% vs. 8.9%; *P* = 0.677) over 12 weeks, but neither difference was statistically significant.

**Table 3:** Secondary outcomes of the study participants at the end of the study

Variables	Intervention (*n* = 45)	Control (*n* = 45)	*P*-value^1^
	Mean ± SD	Mean ± SD	
Waist circumference:			
Baseline	107.76 ± 18.68	102.84 ± 18.31	0.211
2nd follow-up	103.67 ± 16.58	105.46 ± 18.62	0.643
P-value ^2^	0.000*	0.000*	
HbA1c:			
Baseline	9.95 ± 2.92	9.34 ± 2.01	0.254
2nd follow-up	8.12 ± 1.26	10.43 ± 2.21	0.000*
P-value ^2^	0.000*	0.000*	
Total cholesterol:			
Baseline	165.13 ± 67.53	175.76 ± 55.52	0.417
2nd follow-up	135.26 ± 52.62	186.85 ± 54.89	0.000*
P-value ^2^	0.000*	0.000*	
LDL-c:			
Baseline	91.48 ± 55.46	96.97 ± 50.66	0.625
2nd follow-up	71.09 ± 46.66	108.42 ± 51.93	0.001*
P-value ^2^	0.000*	0.000*	
HDL-c:			
Baseline	32.14 ± 12.49	29.92 ± 9.24	0.341
2nd follow-up	39.86 ± 10.25	26.14 ± 8.15	0.000*
P-value ^2^	0.000*	0.000*	
Acute CV events			
2nd follow-up *N (%)*	8 (18.6)	14 (34.1)	0.105
Mortality			
2nd follow-up, N *(%)*	2 (4.4)	4 (8.9)	0.677

1: Independent sample t-test (Comparison between groups)

2: Paired samples t-test (Comparison with baseline)

## Discussion

Adherence to lipid-lowering therapy among patients with diabetes remains a major challenge in Egypt, requiring collective efforts to address. This study provides a valuable and timely contribution to the intersection of healthcare and technology, highlighting the potential of digital interventions.

The findings of this study demonstrated a high level of statin adherence during the initial follow-up period (six weeks after baseline assessment), with both the intervention and control groups showing good adherence based on pill count. While the MARS-5 scores did not show a significant difference, the high adherence rate observed even in the control group may be attributed to the face-to-face health education session provided immediately after the baseline assessment. Such early engagement may have had a positive influence on patients’ motivation and understanding of their medication regimen.

These results are consistent with a previous study [17] which illustrated a high MPR rate (99.9%) among patients with type 2 diabetes in secondary care settings. Although the study design differed, the similarity in outcomes may be due to the shared secondary care setting, where patients typically receive more specialized attention and may be more aware of the risks of poor adherence due to more advanced disease progression and complications. Furthermore, a high prevalence of microvascular and macrovascular complications in both studies likely encouraged adherence.

Contrary to our findings, a recent study [18] found that only one-third of patients with diabetes exhibited high statin adherence. This discrepancy may be explained by differences in the duration of diabetes, their study population included patients with shorter diabetes durations (< 5 years), while our participants had a longer mean duration (9–10 years), potentially leading to increased disease awareness and medication adherence. Similarly, a retrospective study [19] found high discontinuation rates within three months of statin initiation. These contrasting findings may result from differences in study design and follow-up strategy; the retrospective study lacked direct patient engagement or educational interventions, unlike our prospective, interventional approach.

Additionally, a systemic review [9] identified specific adherence obstacles among Egyptian patients, including missed doses, reliance on herbal treatments, and dependence on public health insurance. These issues were similarly reported in previous research [16]. Nevertheless, our intervention group demonstrated enhanced adherence, as evidenced by improvements in both MARS-5 and pill count scores at the second six-week follow-up. This indicates that well-structured educational programs followed by reminder-based strategies can effectively address and mitigate these adherence barriers.

The use of SMS as an adherence-promoting tool appears to be an effective strategy. Comparable results were reported in previous studies [13, 20] which also found improved adherence through tailored text messaging. However, some of these studies were conducted in primary care settings and did not examine secondary outcomes such as changes in lipid profiles. These methodological differences may account for the broader impact observed in the current study.

Moreover, the improved adherence in our intervention group was reflected not only in medication-taking behavior but also in clinical outcomes, including reductions in visceral obesity and HbA1c levels. These improvements may reflect increased patient engagement and lifestyle changes, reinforcing the dual benefit of adherence interventions targeting both pharmacologic and behavioral outcomes.

Interestingly, the study findings revealed statistically significant reduction in the mean total cholesterol and LDL-c levels in the intervention group compared to the control group. This outcome is particularly favorable, as LDL is atherogenic and a major risk factor for cardiovascular diseases. These results align with a recent study [18] which also found lower LDL-C and total cholesterol levels among highly adherent patients. Moreover, our findings support the evidence [21] which demonstrated that statin adherence is associated with reduced endothelial inflammation, lower cardiovascular risk, and fewer hospital admissions.

Although the proportion of patients experiencing acute cardiovascular events and mortality at 12th weeks was lower in the intervention group, the differences were not statistically significant. This is likely due to short follow-up duration and limited sample size, which may have limited the power to detect significant differences. While it is true that significant changes in acute cardiovascular events, and mortality typically require a longer duration to manifest, the inclusion of these secondary outcomes in this study remains relevant. Even within 12th weeks, statin adherence can lead to measurable improvements in lipid profiles, which are early indicators of cardiovascular risk reduction.

Moreover, monitoring acute cardiovascular events and mortality, even within a short time frame, offers meaningful early insights into the possible long-term advantages of adherence interventions. Although more extended follow-up would strengthen these findings, assessing these outcomes at 12th weeks still contributes to understanding the short-term impact of adherence interventions. A comprehensive review [22] highlighted the effectiveness and accessibility of SMS reminders as a digital tool to improve adherence to secondary prevention medications in cardiovascular disease. This strategy is particularly relevant in low- and middle-income countries, where advanced digital health technologies may be limited.

### Limitations

Although more advanced and precise methods for measuring adherence, such as electronic monitoring, exist, their use was not practical in the routine clinical setting of this study. Moreover, the lack of statistically significant association between adherence rates and specific clinical outcomes- as cardiovascular events and mortality-may be explained by the study’s short follow-up period and limited sample size. Additionally, since participants were drawn from an endocrinology outpatient clinic, the results may not fully reflect the broader diabetic population in Egypt, potentially limiting generalizability. Nonetheless, the clinic is part of the oldest university teaching hospital in Upper Egypt and serves a geographically diverse patient base, which adds relevance to the findings.

## Conclusion

Telehealth presents a practical and effective strategy for improving medication adherence and is feasible for implementation across a wide range of geographic settings, including remote areas such as those represented in our study population. While the findings are encouraging, they should be interpreted as preliminary. Future research with larger sample sizes and longer follow-up periods is needed to assess the sustained impact of adherence to lipid-lowering therapy on long-term patient outcomes.

### Implications for policy and practice

Strong evidence supports the effectiveness of SMS in improving statin adherence and clinical outcomes. This study highlights the potential for future research into advanced technologies that aid in patient self-management, particularly among healthcare providers. Healthcare policy systems can also benefit from such interventions, as they optimize time for both providers and patients while making efficient use of limited resources. Integrating information technology-based interventions into undergraduate medical curricula could further enhance their adoption and impact.

## Supplementary Information

Supplementary Material 1.

## Data Availability

The datasets generated and analyzed during the current study will be available from the corresponding author upon reasonable request.

## Abbreviations

CHD: Coronary heart disease
CKD: Chronic kidney disease
CVD: Cardiovascular disease
DKA: Diabetic ketoacidosis
ESRD: End-stage renal disease
HbA1c: High-density lipoprotein
HDL-c: High-density lipoprotein
IHD: Ischemic heart disease
LDL-c: Low-density lipoprotein
MARS-5: Morisky Medication Adherence Scale -5
NCEP-III: National Cholesterol Education Program
PC: Pill count
SMS: Short Messaging Services

## References

[1] Statista. Number of mobile (cellular) subscriptions worldwide from 1993 to 2021. http://www.statista.com/statistics/262950/global-mobile-subscriptions-since-1993.accessed 30 August 2022.

[2] Ministry of Communications and Information Technology. ICT indicators in brief – January 2024.

[3] International Diabetes Federation. IDF Diabetes Atlas. 11th ed. Egypt country profile.; 2025.

[4] World Health Organization. (2023). Cardiovascular diseases (CVDs).https://www.who.int/news-room/fact-sheets/detail/cardiovascular-diseases-(cvds).

[5] Pappan N, Awosika AO, Rehman A, Dyslipidemia. https://www (2025). 32809726

[6] Taylor RS, et al. Global perspectives on heart disease rehabilitation and secondary prevention: a scientific statement from the association of cardiovascular nursing and allied professions, European association of preventive cardiology, and international council of cardiovascular prevention and rehabilitation. Eur Heart J. 2023;44:2515–25. 37477626 10.1093/eurheartj/ehad225 PMC10361025

[7] Chan AHY, Horne R, Hankins M, Chisari C. The medication adherence report scale: a measurement tool for eliciting patients’ reports of nonadherence. Br J Clin Pharmacol. 2020;86:1281–8. 31823381 10.1111/bcp.14193 PMC7319010

[8] ElSayed NA, et al. Cardiovascular Disease and Risk Management: Standards of Care in Diabetes. Diabetes Care. 2024;47:S179–218. 38078592 10.2337/dc24-S010 PMC10725811

[9] Abouzid MR, Ali K, Elkhawas I, Elshafei SM. An overview of diabetes mellitus in Egypt and the significance of integrating preventive cardiology in diabetes management. Cureus. 2022. 10.7759/cureus.27066. 36000101 10.7759/cureus.27066 PMC9390800

[10] Thakkar J, et al. Mobile telephone text messaging for medication adherence in chronic disease: a meta-analysis. JAMA Intern Med. 2016;176:340–9. 26831740 10.1001/jamainternmed.2015.7667

[11] Rea F, Savaré L, Corrao G, Mancia G. Adherence to lipid-lowering treatment by single-pill combination of statin and ezetimibe. Adv Ther. 2021;38:5270–85. 34480293 10.1007/s12325-021-01892-7 PMC8478750

[12] Chioma Ebuenyi M, Schnoor K, Versluis A, Meijer E, Chavannes NH. Short message services interventions for chronic disease management: A systematic review. Clin eHealth. 2021;4:24–9.

[13] Aziz SHA, Monem Sultan E, Ramadan EA, Abd OI. Effect of using smart phone application on Self-Care activities among patients with diabetes mellitus. Egypt Nurs J. 2023;20:147–58.

[14] Schulz KF, Altman DG, Moher D, CONSORT. 2010 statement: updated guidelines for reporting parallel group randomised trials. BMC Med. 2010;8: 18. 20334633 10.1186/1741-7015-8-18 PMC2860339

[15] Masnoon N, Shakib S, Kalisch-Ellett L, Caughey GE. What is polypharmacy? A systematic review of definitions. BMC Geriatr. 2017;17: 230. 29017448 10.1186/s12877-017-0621-2 PMC5635569

[16] Alwhaibi M, et al. Adherence to Statin therapy and attainment of LDL cholesterol goal among patients with type 2 diabetes and dyslipidemia. Patient Prefer Adherence. 2019;13:2111–8.<\/p> 31853174 10.2147/PPA.S231873 PMC6916674

[17] Beernink JM, et al. Adherence to statin therapy and attainment of LDL cholesterol targets in an outpatient population of type 2 diabetes patients: analysis in the diabetes and lifestyle cohort Twente (DIALECT). Front Pharmacol. 2022. 10.3389/fphar.2022.888110. 35903346 10.3389/fphar.2022.888110 PMC9315395

[18] Alharbi MS, et al. Adherence to Statin Among Diabetic Patients in Diabetic Centers in Qassim Region, Saudi Arabia. *Cureus.* 2023. https://doi.org/10.7759/cureus.46742. 10.7759/cureus.46742 PMC10631564 38022032

[19] Zhao B, He X, Wu J, Yan S. Adherence to statins and its impact on clinical outcomes: a retrospective population-based study in China. BMC Cardiovasc Disord. 2020;20: 282. 32522146 10.1186/s12872-020-01566-2 PMC7288497

[20] Kassavou A, et al. A highly tailored text and voice messaging intervention to improve medication adherence in patients with either or both hypertension and type 2 diabetes in a UK primary care setting: feasibility randomized controlled trial of clinical effectiveness. J Med Internet Res. 2020;22:e16629. 32427113 10.2196/16629 PMC7267991

[21] Sørensen AL, Hasselbalch HC, Nielsen CH, Poulsen HE, Ellervik C. Statin treatment, oxidative stress and inflammation in a Danish population. Redox Biol. 2019;21:101088. 30594900 10.1016/j.redox.2018.101088 PMC6307042

[22] Adler AJ, et al. Mobile phone text messaging to improve medication adherence in secondary prevention of cardiovascular disease. Cochrane Database Syst Reviews. 2017. 10.1002/14651858.CD011851.pub2 PMC6478182 28455948

